# 

**Published:** 1851-11

**Authors:** 


### CONTENTS:

### PART I.—ORIGINAL COMMUNICATIONS.

ART. 1.—Observations on Cerebro-Arachnitis, or Miningitis, by James Smick, M. D. 234
ART. 2.—Singular Influence of the Aurora Borealis, by R. S. Miller, Esq. 253
ART. 3.—Occlusion of the Os Uteri, by E. G. Mygatt, M. D. 255
ART. 4.—Collodion in Small Pox and other Cutaneous Diseases, by E. McArthur, M. D., 358
ART. 5.—Extract of Beefs' Blood in the treatment of Anæmia, by Dr. Von Mauthner of Vienna. 261
ART. 6.—Rubeola, Complicated with premature Labor, by O. Pope Hatheway, M. D. 262
ART. 6.—Meeting of the Physicians of Whitesides County, Illinois. 264
ART. 7.—Clinical Lecture in the Medical Ward of the Illinois General Hospital, by N. S. Davis, M. D. 265

### PART II.—REVIEWS AND NOTICES OF NEW WORKS.

ART. 1—Proceedings of the Illinois State Medical Society. 272
ART. 2.—On the Theory and Practice of Midwifery, by F. Churchill, M. D. 280
ART. 3.—A Practical Treatise on the Disease and Injuries of the Uniary Bladder, Prostrate Gland and Urethra, by S. D. Gross, M. D. 281
ART. 4.—The laws of Health in relation to Mind and Body: A series of letters from an old practitioner to the patient, by L. J. Beale, M. D. 282
ART. 5.—Letters to a candid inquirer on Animal Magnetism, by William Gregory, M. D., F. R. S. C. 283

### PART III.—SELECTIONS.

ART. 1.—Remarks on Digestion, by N. P. Lattimore, M. D. 285
ART. 2.--Diameters of the Fœtal head, from measurements made in the Dublin Lying-in Hospital, by Addinell Hewson, M. D. 290
ART. 3.--Case of Hermaphroditism, by Dr. John Neill, 293
ART. 4.—Extracts from the Records of the Boston Society for Medical Improvement, by William W. Moreland, 295
ART. 5.—An Ovary removed by mistake for a Libial Cyst. 297

### PART IV.—EDITORIAL.

ART. 1.—Prof. Davis' Introductory Lecture at the opening of Rush Medical College, 298
ART. 2.—The Journal, 303
ART. 3.—Miscellaneous Medical Intelligence, 304
Obituary, 306
ART. 4.—Notice to Readers and Correspondents. 307

